# Dr. William Waring Johnston

**Published:** 1902-04

**Authors:** 


Dr. William Waring Johnston, of Washington, D. C., son of the late Dr. William P. Johnston, died at Atlantic City, N. J., March 22, 1902, aged 58 years. He graduated from the University of Pennylvania, March, 1865, and after spending three years abroad established himself in practice in his native city. He was famous as a diagnostician, as his father before him had been, and his patients numbered some of the most distinguished men in public life during the last thirty years.

